# Supplementary material for: Microspectroscopic visualization of how biochar lifts the soil organic carbon ceiling
Source: Nat Commun. 2022 Sep 2;13:5177. doi: 10.1038/s41467-022-32819-7 (PMC9440262; doi:10.1038/s41467-022-32819-7)
Supplement: Supplementary file 1 — Supplementary Information [file 41467_2022_32819_MOESM1_ESM.pdf]

Supplementary Information

Microspectroscopic visualization of how biochar lifts the soil organic carbon ceiling

Zhe (Han) Weng[1,2,3,4], Lukas Van Zwieten[1,5★], Ehsan Tavakkoli[6,7], Michael T. Rose[1], Bhupinder Pal Singh[2], Stephen Joseph[8,9], Lynne M. Macdonald[10], Stephen Kimber[1], Stephen Morris[1], Terry J. Rose[5], Braulio S Archanjo[11], Caixian Tang[3], Ashley E. Franks[12,13], Hui Diao[14], Steffen Schweizer[15], Mark J. Tobin[16], Annaleise R. Klein[16], Jitraporn Vongsvivut[16], Shery L. Y. Chang[17], Peter M. Kopittke[4], Annette Cowie[2,18]

[1]NSW Department of Primary Industries, Wollongbar Primary Industries Institute, Wollongbar, NSW 2477, Australia
[2]School of Environmental and Rural Science, University of New England, Armidale, NSW 2351, Australia
[3]Department of Animal, Plant & Soil Sciences, Centre for AgriBioscience, La Trobe University, Melbourne, VIC 3086, Australia
[4]School of Agriculture and Food Sciences, The University of Queensland, St. Lucia, QLD 4072, Australia
[5]Southern Cross University, East Lismore, NSW 2480, Australia
[6]NSW Department of Primary Industries, Wagga Wagga Agriculture Institute, Wagga Wagga, NSW 2650, Australia
[7]School of Agriculture, Food & Wine, The University of Adelaide, Glen Osmond SA 5064, Australia
[8]Institute for Superconducting and Electronic Materials and School of Physics, University of Wollongong, NSW 2522, Australia
[9]School of Materials Science and Engineering, University of New South Wales, Sydney, NSW 2052 Australia
[10]CSIRO Agriculture & Food, Waite campus, Glen Osmond, SA 5064, Australia
[11]Materials Metrology Division, National Institute of Metrology, Quality and Technology (INMETRO), Rio de Janeiro, 25250−020, Brazil
[12]Department of Physiology, Anatomy and Microbiology, La Trobe University, Melbourne, VIC 3086, Australia
[13]Centre for Future Landscapes, La Trobe University, Melbourne, VIC 3086, Australia
[14]Centre for Microscopy and Microanalysis, The University of Queensland, QLD, 4072, Australia
[15]School of Life Sciences, Technical University of Munich, Germany
[16]Australian Nuclear Science and Technology Organisation (ANSTO), Australian Synchrotron, Clayton, VIC 3168, Australia
[17]Electron Microscope Unit, Mark Wainwright Analytical Centre and School of Materials Science and Engineering, University of New South Wales, Sydney, NSW 2052 Australia
[18]NSW Department of Primary Industries, Armidale, NSW 2351, Australia
★e-mail: lukas.van.zwieten@dpi.nsw.gov.au

Supplementary Table 1 | Four fresh field soil types with different biochar amendments over 9.5 years of trials (established in 2006). The rate of biochar application for both amendments was 10 Mg ha^{-1}. Pasture management (ryegrass sowing, NPK application, and weed control) was the same at all sites and followed Slavich et al. (2013).

Treatment	Description
Control	Pasture managed since 2006 with no addition of biochar
Historical	Biochar applied at trial establishment in 2006
Control+Recent	Biochar added into Control plots at 8.2 years after trial establishment
Historical+Recent	Biochar added into Historical plots at 8.2 years after trial establishment

Supplementary Table 2 | Contribution of root respiration to total CO_2 fluxes during each pulse labelling event in three treatments. Values ± SE (n=3).

Treatment	Contribution of root respiration to total CO_2 fluxes (%)		
	8.9 years	9.2 years	9.5 years
Control	30.8 ± 3.2	28.4 ± 2.8	31.6 ± 4.1
Control+Recent	29.5 ± 2.9	29.5 ± 1.3	35.5 ± 5.3
Historical+Recent	30.7 ± 2.3	27.1 ± 1.8	27.6 ± 3.4

Supplementary Table 3 | Proportion of belowground ^{13}C recovered from soil+root respiration, root biomass, and bulk soil after pulse labelling events at 8.9, 9.2, and 9.5 y after trial establishment in two treatments. Values given as percentage of total applied ^{13}C ± SE, n=3. * $P< 0.05$, two-way ANOVA, relative to Control+Recent data.

	Pulse labelling at	8.9 years		9.2 years		9.5 years	
		Control+Recent	Historical+Recent	Control+Recent	Historical+Recent	Control+Recent	Historical+Recent
Soil+Root respiration	(%)	4.53 ± 0.13	3.80 ± 0.15	1.14 ± 0.08	1.85 ± 0.34	3.99 ± 0.18	3.26 ± 0.19
Root biomass	(%)	27.32 ± 3.91	27.52 ± 3.68	17.22 ± 7.46	27.03 ± 1.43*	9.12 ± 2.30	10.92 ± 2.51
Bulk soil	(%)	7.64 ± 5.23	12.01 ± 3.37	17.62 ± 15.7	23.51 ± 15.23	32.14 ± 3.04	44.13 ± 4.03*
Sum	(%)	39.51 ± 9.27	43.32 ± 7.20	36.01 ± 23.24	52.32 ± 17.03	45.23 ± 4.52	58.21 ± 5.74*

Supplementary Table 4 | Proportion of belowground ^{13}C recovered from free particulate organic matter (F-POM), occluded particulate organic matter (O-POM), and mineral-protected soil organic matter (M-SOM) fractions after a pulse labelling event at 9.5 y after trial establishment in two treatments. Values given as percentage of total applied $^{13}C \pm$ SE, n=3. * P< 0.05, two-way ANOVA, relative to Recent + Control data.

	Control+Recent	Historical+Recent
F-POM	2.74 ± 1.92	2.73 ± 1.34
O-POM	21.81 ± 3.80	20.22 ± 2.80
M-SOM	7.60 ± 0.28	21.13 ± 3.86*

Supplementary Table 5 | Microbial biomass carbon (MBC), and metabolic quotient of both total SOC and rhizodeposit-derived C in two treatments. Values given ± SE, n=3.

	8.9 years		9.2 years		9.5 years	
	Control+ Recent	Historical+ Recent	Control+ Recent	Historical+ Recent	Control+ Recent	Historical+ Recent
Microbial biomass C (mg g^{-1} dry soil)	1.41 ± 0.10	1.26 ± 0.04	1.45 ± 0.07	1.33 ± 0.13	1.46 ± 0.33	1.35 ± 0.29
Metabolic quotient of bulk SOC (µg CO_2-C mg^{-1} MBC h^{-1})	1.60 ± 0.03	1.27 ± 0.08	0.81 ± 0.03	0.66 ± 0.07	0.34 ± 0.04	0.23 ± 0.04
Metabolic quotient of root-derived C (µg CO_2-^{13}C g^{-1} MBC h^{-1})	1.48 ± 0.12	1.04 ± 0.39	0.13 ± 0.06	0.39 ± 0.03	0.16 ± 0.03	0.11 ± 0.02

Supplementary Table 6 | The specific enzyme activities within the soil+root system of three treatments. Values shown the ratio of enzyme activity (MUB) to total microbial biomass (MBC) ± SE, n=3.

Specific enzyme activity (nmol MUB mg^{-1} MBC h^{-1})	Control	Control+ Recent	Historical+ Recent
ß-glucosidase	174 ± 32	133 ± 28	167 ± 56
Xylosidase	47 ± 5	34 ± 4	37 ± 4
Cellulase	35 ± 10	26 ± 12	26 ± 6
N-acetyl-glucosaminidase	119 ± 23	98 ± 23	97 ± 15
Phosphatase	609 ± 129	418 ± 52	322 ± 58

Supplementary Table 7 | Enzyme activities within the soil only and soil+root systems in two treatments. No pH effect by biochar amendment was detected. Values given as enzyme activity (MUB) per unit soil or soil+ root mass ± SE, n= 24. * P< 0.05, two-way ANOVA, relative to activity in Control soils).

	Soil only		Soil+root	
Enzyme activity (nmol MUB g^{-1} soil h^{-1})	Control+ Recent	Historical+ Recent	Control+ Recent	Historical+ Recent
ß-glucosidase	-45.32 ± 6.59*	-16.6 ± 7.89*	0.56 ± 16.1	31.52 ± 32.2
Xylosidase	-22.13 ± 2.94*	-13.2 ± 1.29*	-3.00 ± 2.11	-1.83 ± 2.27
Cellulase	-7.90 ± 3.08*	-5.60 ± 4.20*	-1.28 ± 6.94	-2.64 ± 3.15
N-acetyl-glucosaminidase	-47.81 ± 8.00*	-24.02 ± 12.2	10.32 ± 13.18	0.28 ± 8.61
Phosphatase	-209 ± 12.6*	-149 ± 18.8*	-64 ± 29.9	-222 ± 33.2*

Supplementary Table 8 | Standard curve fits for three treatments to account for potential quenching or excitation of the fluorophore in enzyme activity analysis. Standard errors of coefficient estimates (n=3) are for intercept and slope (where the x-axis is methylumbelliferyl concentration [µM] and y-axis is relative fluorescence).

Treatment	Intercept	Slope
Control	971 ± 290	168 ± 3
Historical+Recent	924 ± 321	169 ± 3
Control+Recent	765 ± 236	168 ± 2

Supplementary Table 9 | The major and functional groups in the hardwood biochar before incorporation into the soil and after 1 year. X-ray photoelectron spectroscopy (XPS) of both whole and crushed (< 50 µm) 1.3-year biochar particles were undertaken and compared with the fresh biochar. The values of the binding energies (B.E.), atomic percentage (%), and the O/C ratio were obtained for 5 different components (n=3).

			Fresh biochar		1.3-year biochar		Crushed 1.3-year biochar	
Component	Likely bonds		B.E. (eV)	%	B.E. (eV)	%	B.E. (eV)	%
C1	C=C, C-C, C-H	C *sp*^{2}	284.6	75	284.6	23	284.6	61
C2	C-OH, C-O-C, C-N	C *sp*^{3}	286.0	12	286.1	30	286.1	19
C3	C=O		287.2	3	287.2	28	287.4	10
C4	COOH		288.8	8	289.0	14	289.1	7
C5		$\pi \rightarrow \pi^*$ transition of delocalized *sp*^{2} electrons	291.5	2	291.0	5	291	3

Supplementary Table 10 | The concentrations of dissolved organic carbon (DOC) and its fractions measured by LC-OCD. CDOC, hydrophilic chromatographable organic carbon that elutes from the column; HOC, hydrophobic organic carbon fraction that binds irreversibly to the solid phase of the column. The CDOC fraction is further sub-divided into five categories based on retention time, including bio-polymers, persistent C substances, building blocks (oxidized persistent C including polyaromatic acids and polyphenols), low molecular weight (LMW) neutrals, and LMW acids (not quantified). Values given ± SE, n=3.* P < 0.05 by *t*-test.

C fractions (mg g^{-1})	Control+ Recent	Historical+ Recent
DOC	0.90 ± 0.03	1.31 ± 0.02*
HOC	0.09 ± 0.002	0.11 ± 0.003*
CDOC	0.81 ± 0.18	0.81 ± 0.11
Biopolymers	0.20 ± 0.02	0.22 ± 0.01
Persistent C	0.40 ± 0.07	0.35 ± 0.07
Building blocks	0.11 ± 0.03	0.21 ± 0.02*
LMW neutrals	0.12 ± 0.04	0.10 ± 0.05
Aromaticity	1.46 ± 0.11	1.52 ± 0.37

Supplementary Table 11 | Deconvolution and peak fitting of the double normalized SXR spectra. The relative proportion of functional groups, peak height, peak position, full width at half maximum (FWHM) and peak area were calculated using in-house script on Matlab. Measures of the goodness of fitting using R^2 errors of better than 0.999 were achieved for the data.

		Control+Recent mineral	Historical+Recent mineral	Conttrol+Recent microaggregate	Historical+Recent microaggregate	Recent biochar	Historical biochar
Relative proportion of functional groups (%)	Aromatic & quinone C	n.a.	n.a.	24.1	19.1	15.5	12.2
	Aromatic C	18.5	11.1	9.7	10.8	35.7	33.8
	Aromatic C substituted	n.a.	n.a.	6.8	5.8	21.4	19.5
	Aliphatic C	15.6	30.6	31.9	33.0	21.3	23.9
	Carboxylic C	65.8	58.3	27.5	31.3	6.1	10.6
Peak Height (a.u.)	Aromatic & quinone C	n.a.	n.a.	0.58	0.4325	0.4845	0.3834
	Aromatic C	0.2563	0.1845	0.3321	0.2957	0.9418	0.8214
	Aromatic C substituted	n.a.	n.a.	0.1638	0.1226	0.5645	0.4617
	Aliphatic C	0.3075	0.8299	0.8503	0.8041	0.56	0.6475
	Carboxylic C	1.587	1.859	1.043	1.052	0.3208	0.5256
Peak position (eV)	Aromatic & quinone C	n.a.	n.a.	284	283.9	284.2	284
	Aromatic C	285.1	285.2	284.8	284.8	285	284.8
	Aromatic C substituted	n.a.	n.a.	286.6	286.6	286.1	286.1
	Aliphatic C	287.3	287.3	287.3	287.3	287.2	287.2
	Carboxylic C	288.5	288.5	288.4	288.4	288.4	288.5
FWHM (eV)	Aromatic & quinone C	n.a.	n.a.	0.9271	0.8758	0.9909	0.888
	Aromatic C	1.0286	1.0929	0.6551	0.7255	1.1765	1.1499

	Aromatic C substituted	n.a.	n.a.	0.9306	0.9419	1.1772	1.1772
	Aliphatic C	0.7239	0.6718	0.8383	0.8136	1.1772	1.0326
	Carboxylic C	0.5906	0.5708	0.5892	0.5915	0.5871	0.5637
	Aromatic & quinone C	n.a.	n.a.	0.5723	0.4032	0.511	0.3624
	Aromatic C	0.2806	0.2147	0.2316	0.2284	1.1794	1.0054
Peak area (a.u. * eV)	Aromatic C substituted	n.a.	n.a.	0.1623	0.1229	0.7073	0.5785
	Aliphatic C	0.2369	0.5934	0.7588	0.6963	0.7018	0.7117
	Carboxylic C	0.9978	1.1294	0.6543	0.6621	0.2004	0.3154
	sse	0.0626	0.1257	0.0097	0.0105	0.0044	0.0057
	rsquare	0.9994	0.9989	0.9999	0.9999	0.9998	0.9998
Goodness of fitting	dfe	64	64	58	58	53	53
	adjrsquare	0.9992	0.9987	0.9998	0.9998	0.9997	0.9997
	rmse	0.0313	0.0443	0.0129	0.0135	0.0091	0.0104

Supplementary Table 12 | Normalized optical proportions of different features from the IRM maps from two treatments. Values for each feature were combined into mixed categories: a polysaccharide (poly) category of co-localized segments of polysaccharide and aromatic; a mixed aromatic category including the aromatic high intensity regions; and various aliphatic and clay categories including combinations with other channels. The IRM analysis was conducted using triplicate soil samples (i.e., three maps per soil type, see Fig. 5b).

		Poly	Poly+ aromatic	Aliphatic	Aliphatic+ clay	Poly+ aliphatic	Aromatic+ aliphatic	Poly+ aromatic+ aliphatic	Clay	Poly+ clay	Aromatic+ clay	Poly+ aromatic+ clay	Poly+ aliphatic+ clay	Aromatic	Aromatic+ aliphatic+ clay
Mineral fraction	Control+ Recent	30.4 ± 3.1	11.1 ± 2.9	n.a.	17.2 ± 0.7	2.6 ± 0.8	5.2 ± 1.6	2.1 ± 0.2	2.3 ± 0.1	5 ± 1.6	1.5 ± 0.6	n.a.	3.4 ± 0.5	1.7 ± 0.4	17.5 ± 1.4
	Historical+ Recent	23 ± 2.6	13 ± 1.2	0.5 ± 0.2	22.4 ± 0.2	3.1 ± 0.4	5.8 ± 1.0	1 ± 0.4	1.6 ± 0.8	6 ± 1.5	2.4 ± 1.2	n.a.	4 ± 1.3	6 ± 0.9	11.2 ± 2.2
Microaggregate	Control+ Recent	2 6 ± 7.2	10.9 ± 2.9	0.9 ± 0.5	11.7 ± 5.6	3.9 ± 0.2	4.4 ± 1.1	2.6 ± 0.2	4.8 ± 2.2	4.8 ± 1.4	7.2 ± 0.3	0.2 ± 0.2	0.5 ± 0.2	1.9 ± 0.2	20 ± 1.4
	Historical+ Recent	34.9 ± 2.3	6 ± 0.7	0.3 ± 0.3	10.7 ± 3.8	3.3 ± 0.6	2.9 ± 1.4	0.7 ± 0.4	1.8 ± 0.6	4.3 ± 0.6	2 ± 0.5	n.a.	2 ± 0.4	2.4 ± 1.0	28.7 ± 4.5
Significance															
Aggregate		0.479	0.406	0.143	0.034	0.259	0.141	0.613	0.296	0.392	0.006	0.357	0.011	0.012	0.006
Treatment		0.753	0.175	0.495	0.528	0.981	0.885	0.001	0.166	0.694	0.014	0.604	0.201	0.003	0.66
Aggregate × Treatment		0.117	0.447	0.245	0.370	0.289	0.329	0.329	0.373	0.441	0.004	0.377	0.527	0.008	0.024

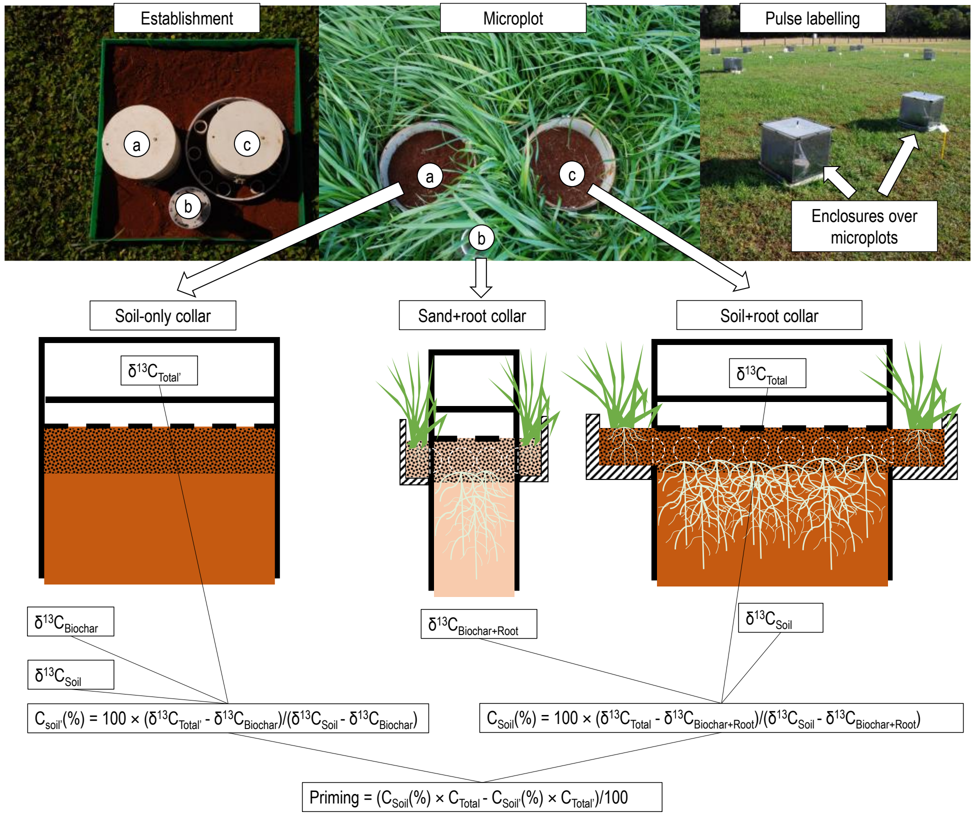

Supplementary Figure 1 | Experimental set-up for microplots, showing how ^{13}C carbon signatures were derived and used to calculate soil mineralization and total soil priming. One microplot showing the experimental layout at plot establishment, after respiration measurement, and during pulse labelling (top). A cross section of contents is shown for (a) soil-only respiration, (b) sand+root+biochar, and (c) soil+root respiration collars in a Control+Recent microplot (bottom). The area outside the respiration collars is planted with an annual ryegrass. $\delta^{13}C_{Total}$, $\delta^{13}C$ signature of total respiration from the soil+root collars after pulse labelling; $\delta^{13}C_{Total'}$, $\delta^{13}C$ signature of total respiration from the soil-only collar after pulse labelling; $\delta^{13}C_{Biochar+Root}$, $\delta^{13}C$ signature of total respiration from the sand+root+biochar collar after pulse labelling; $\delta^{13}C_{Soil}$, $\delta^{13}C$ signature of the soil-derived respiration from soil-only Control collars prior to pulse labelling; $\delta^{13}C_{Biochar}$, $\delta^{13}C$ signature of biochar; C_{Soil}, mineralization of SOC in soil with plant growth; $C_{Soil'}$, mineralization of SOC in soil with no plant growth

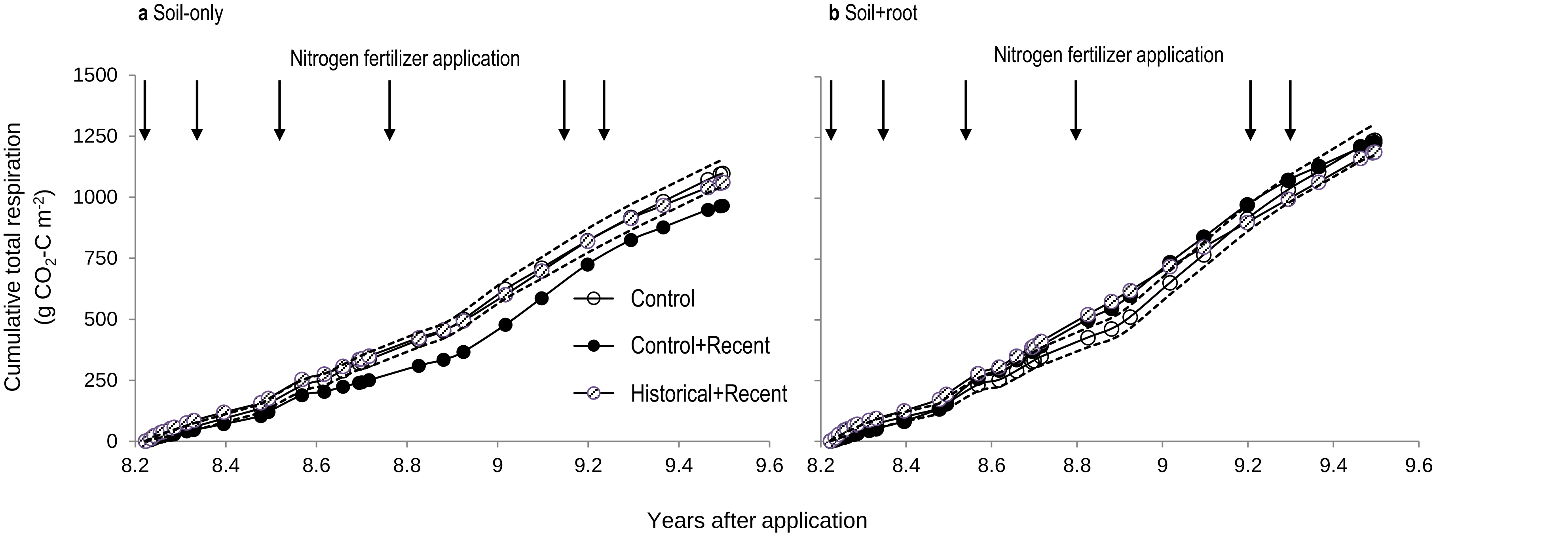

Supplementary Figure 2 | Cumulative total CO_2-C fluxes in three treatments in (a) soil-only and (b) soil+root systems. Confidence intervals (95%) are plotted using dashed lines for Control soils and normalized against the mean squares across all treatments (n=3).

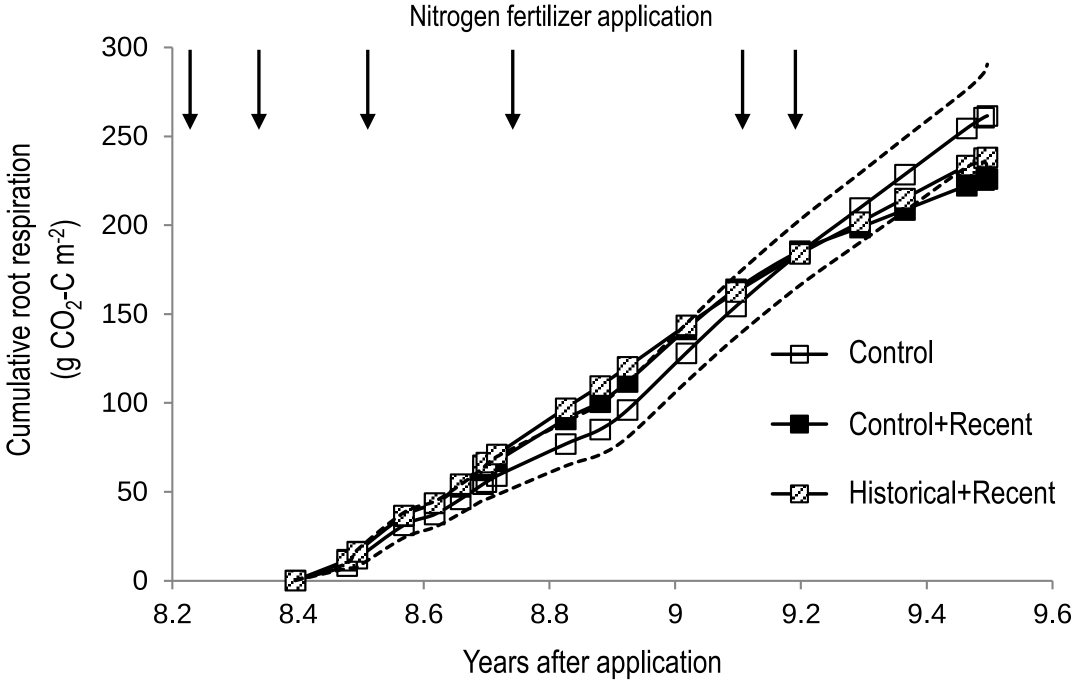

Supplementary Figure 3 | Cumulative root respiration in three treatments. Confidence intervals (95%) are plotted using dashed lines for Control soils and normalized against the mean squares across all treatments (n=3).

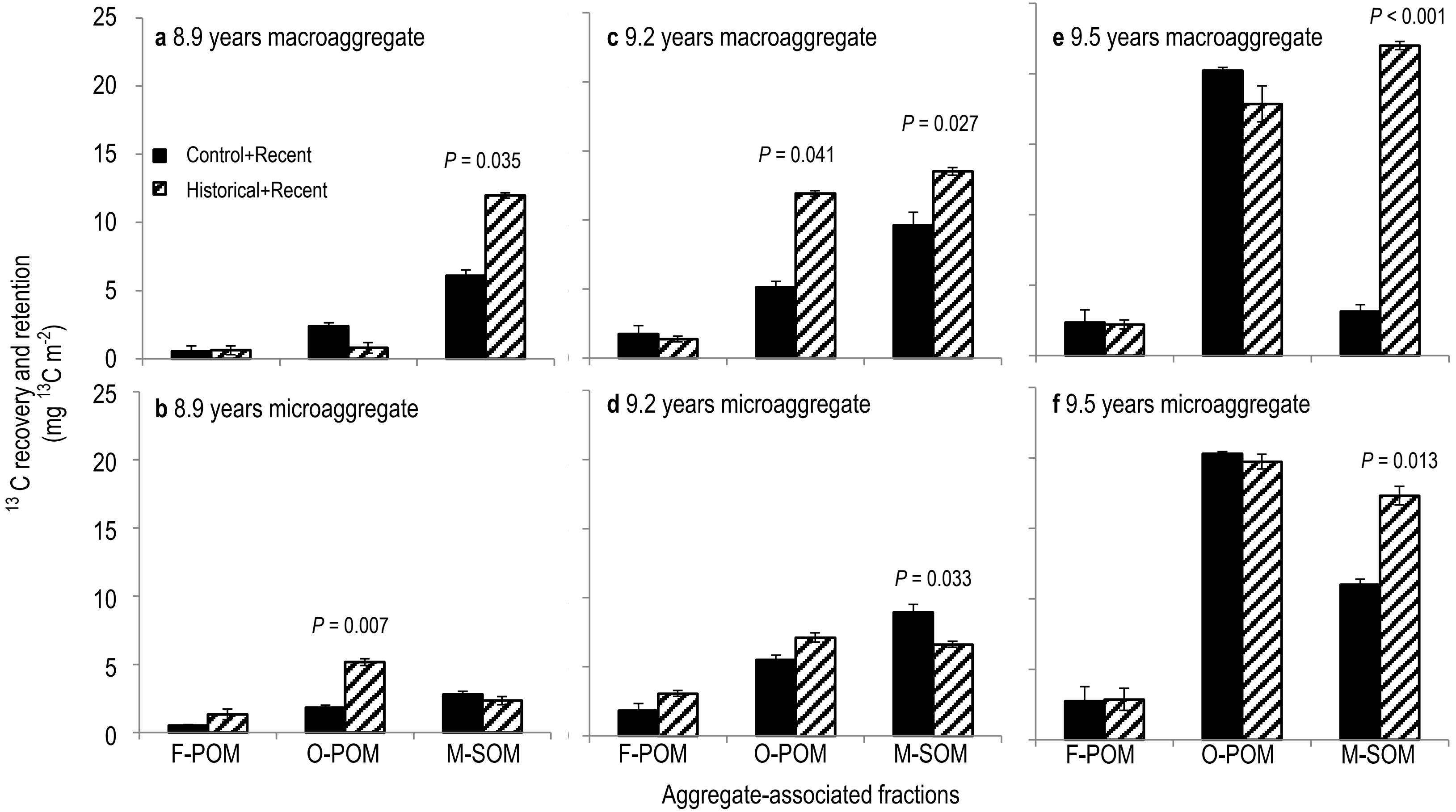

Supplementary Figure 4 | Belowground ^{13}C recovery and retention (0–100 mm) in different C pools in soil macroaggregates (250–2000 μm, a,c,e) and microaggregates (53–250 μm, b,d,f) sampled on Day 15 following the 8.9-, 9.2- and 9.5-year pulse-labelling events. The same amount of ^{13}C enrichment (190 mg m^{-2}) was allocated to each microplot. F-POM, free particulate organic matter (ρ < 1.6 kg m^{-3}); O-POM, occluded particulate organic matter (ρ < 1.6 kg m^{-3}, size >53 μm); M-SOM, mineral-protected soil organic matter (ρ < 1.6 kg m^{-3}, size <53 μm). Values ± SE bars (n=3). * P< 0.05, stats test.

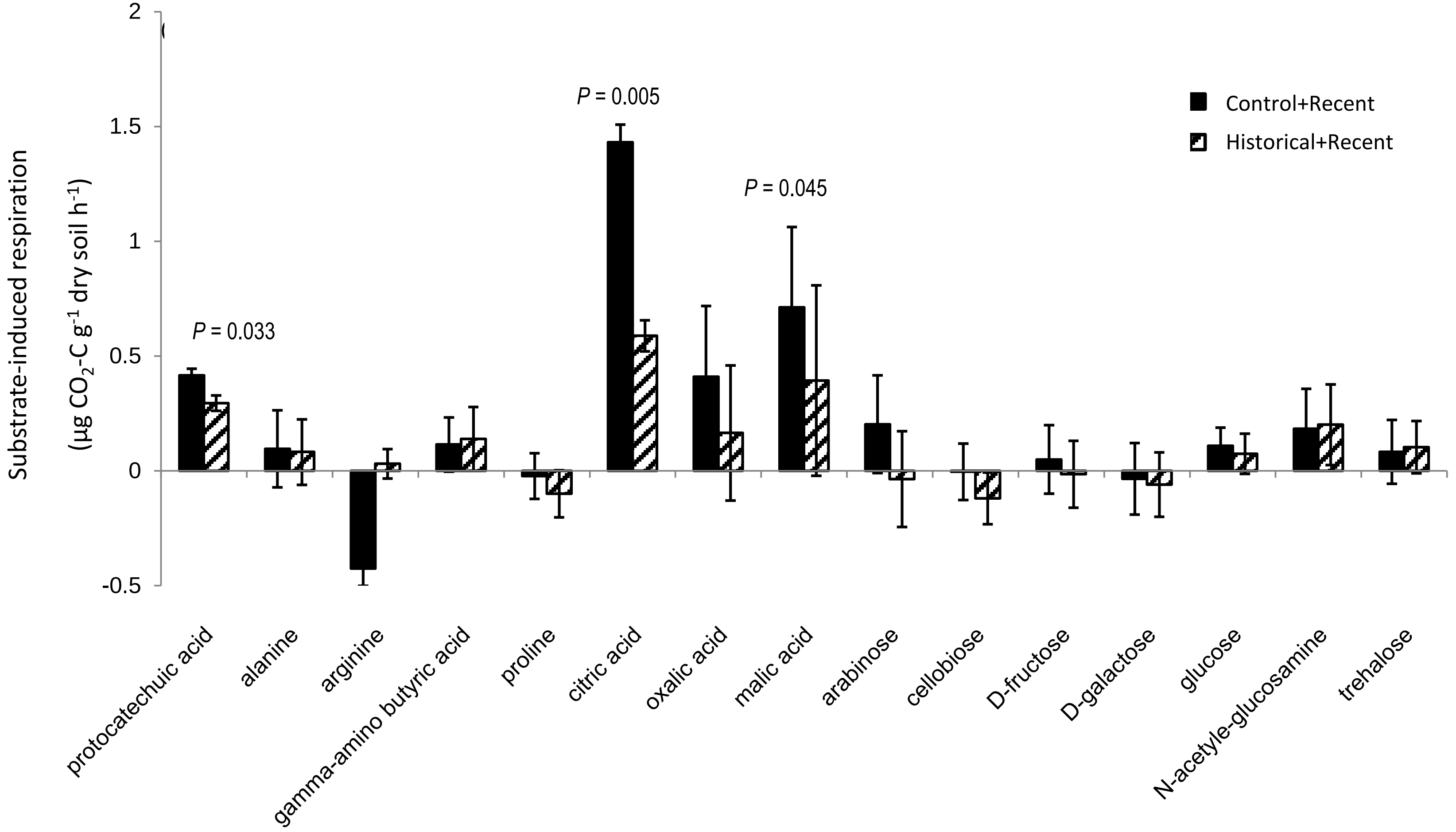

Supplementary Figure 5 | Differences in substrate-induced respiration between the Control vs Recent or Historical+Recent soils. No pH effect from biochar amendments was detected. Values ± SE bars (n=24). GlcNAc, N-acetyl-glucosamine; GABA, γ-amino butyric acid. * P< 0.05.

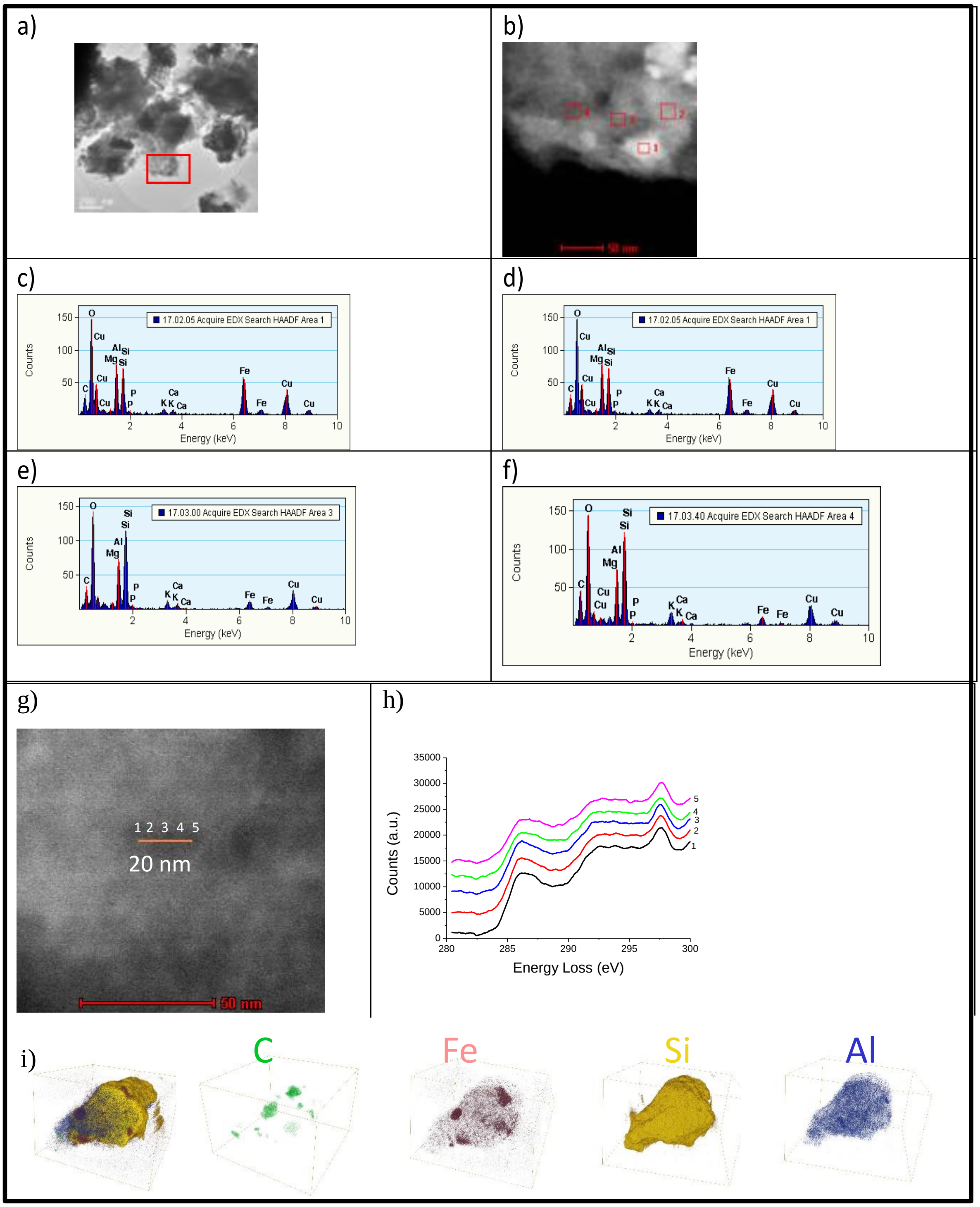

Supplementary Figure 6 | Imaging and analysis of an organo-mineral cluster from Control+Recent soil. a,**b** HAADF image of an organo-mineral cluster. Box in **a** shows the region imaged in **b**. **c**,**d**,**e**,**f**, EDS spectra of regions 1–4 in **b**. **g**,**h** HAADF image (**g**) and EELS spectrum (**h**) of the boxed area. **i**, Elemental composition of an aggregate from Control soil (3D-FIB-SEM-EDS).

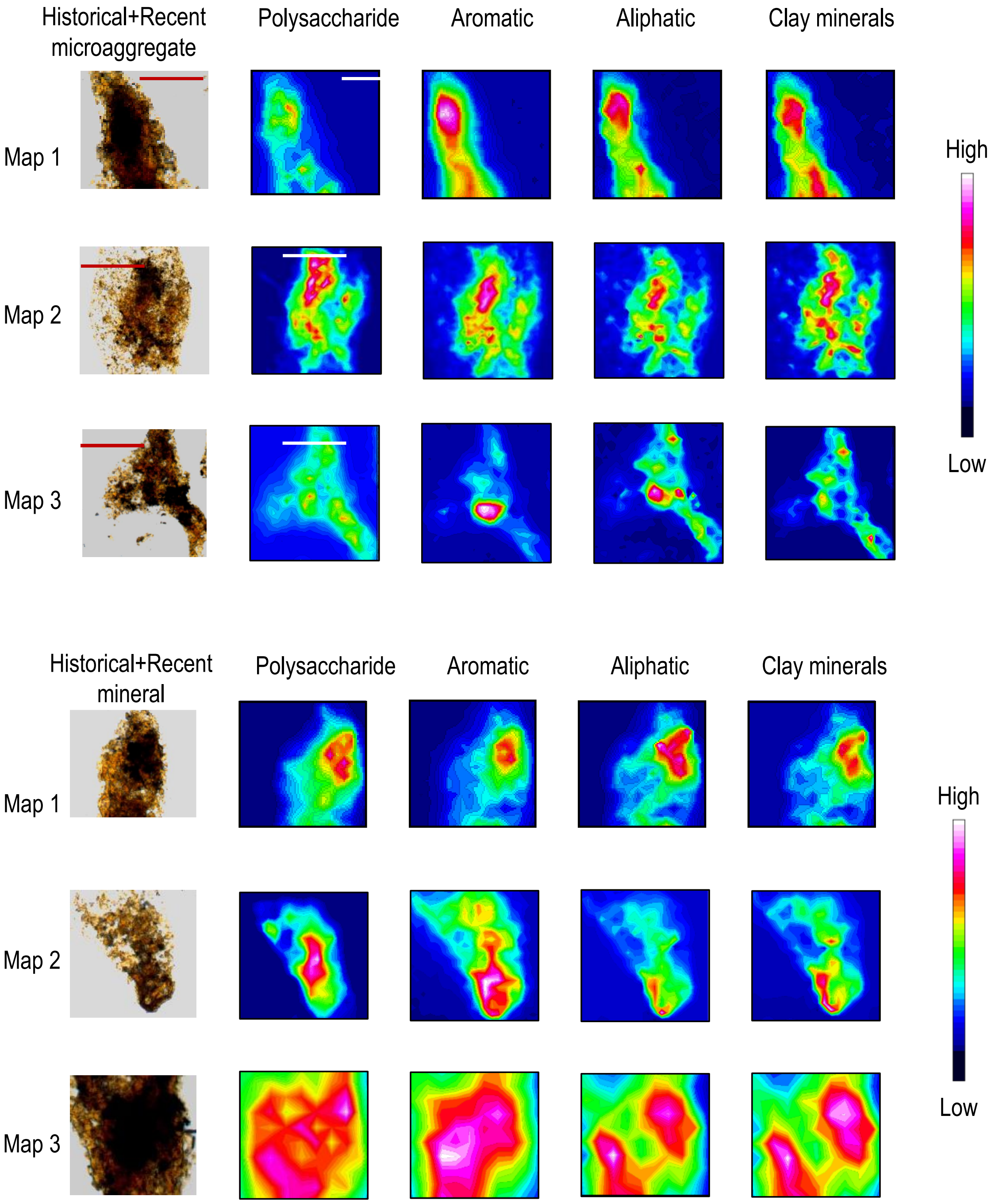

Supplementary Figure 7 | Synchrotron-based infrared microspectroscopy (IRM) of semi-thin (200 nm) sections of free water-stable microaggregates (53–250 µm) and mineral fractions (<53 µm) from Historical+Recent soils. Spectral maps show the distribution of polysaccharide-C (1035 cm^{-1}), aromatic-C (1600 cm^{-1}), aliphatic-C (2920 cm^{-1}), and mineral-OH (3650 cm^{-1}) obtained from 64 co-added scans (4 cm^{-1} resolution), at 5 µm lateral resolution. Images on the left are optical micrographs of the semi-thin sections at the same scale as spectral images. Bars, 50 µm.

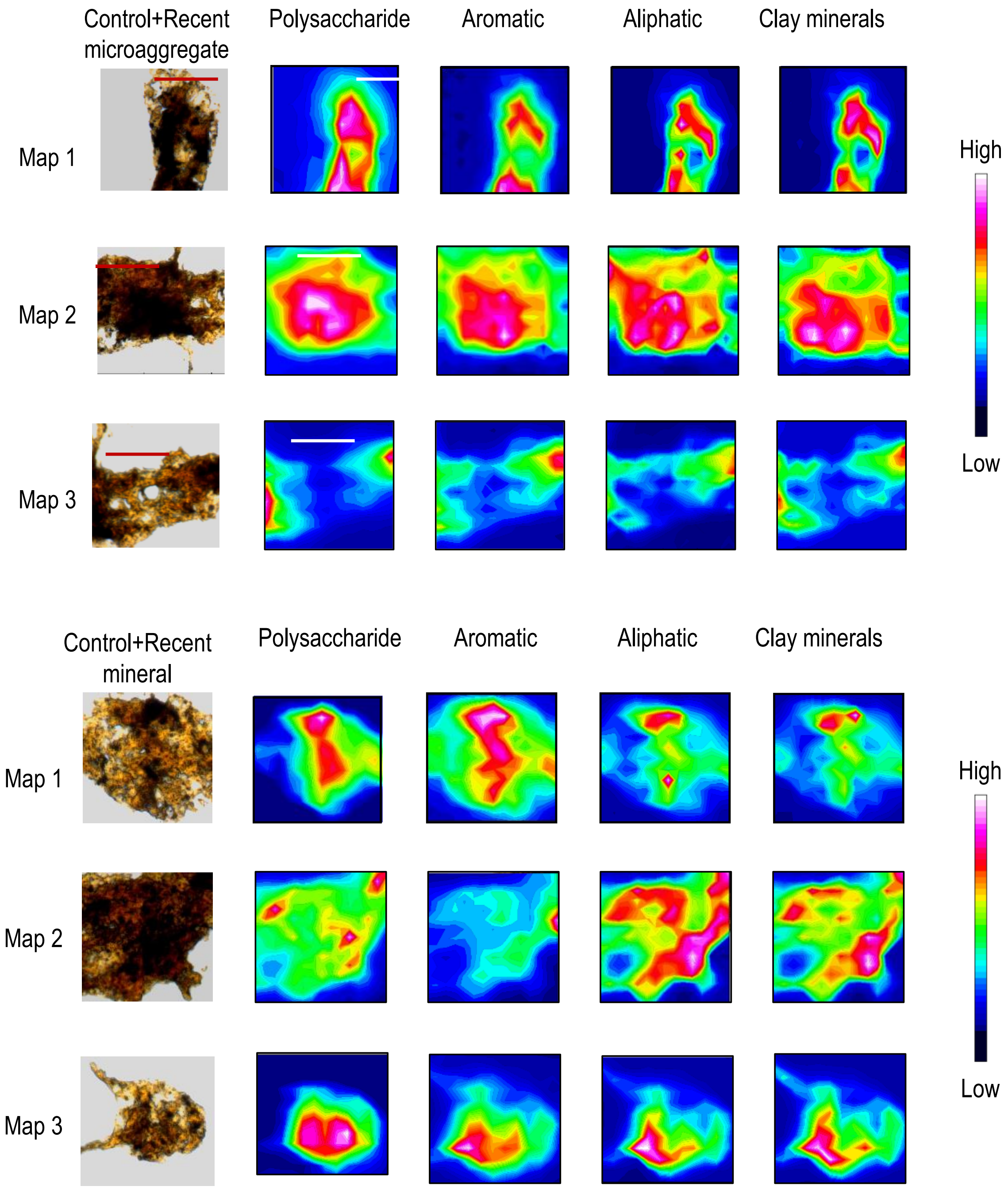

Supplementary Figure 8 | Synchrotron-based IRM of semi-thin (200 nm) sections of free water-stable microaggregates (53–250 µm) and mineral fractions (<53 µm) from Control+Recent soils. Spectral maps show the distribution of polysaccharide-C (1035 cm^{-1}), aromatic-C (1600 cm^{-1}), aliphatic-C (2920 cm^{-1}), and mineral-OH (3650 cm^{-1}) obtained from 64 co-added scans (4 cm^{-1} resolution), at 5 µm lateral resolution. Images on the left are optical micrographs of the semi-thin sections at the same scale as spectral images. Bars, 50 µm.

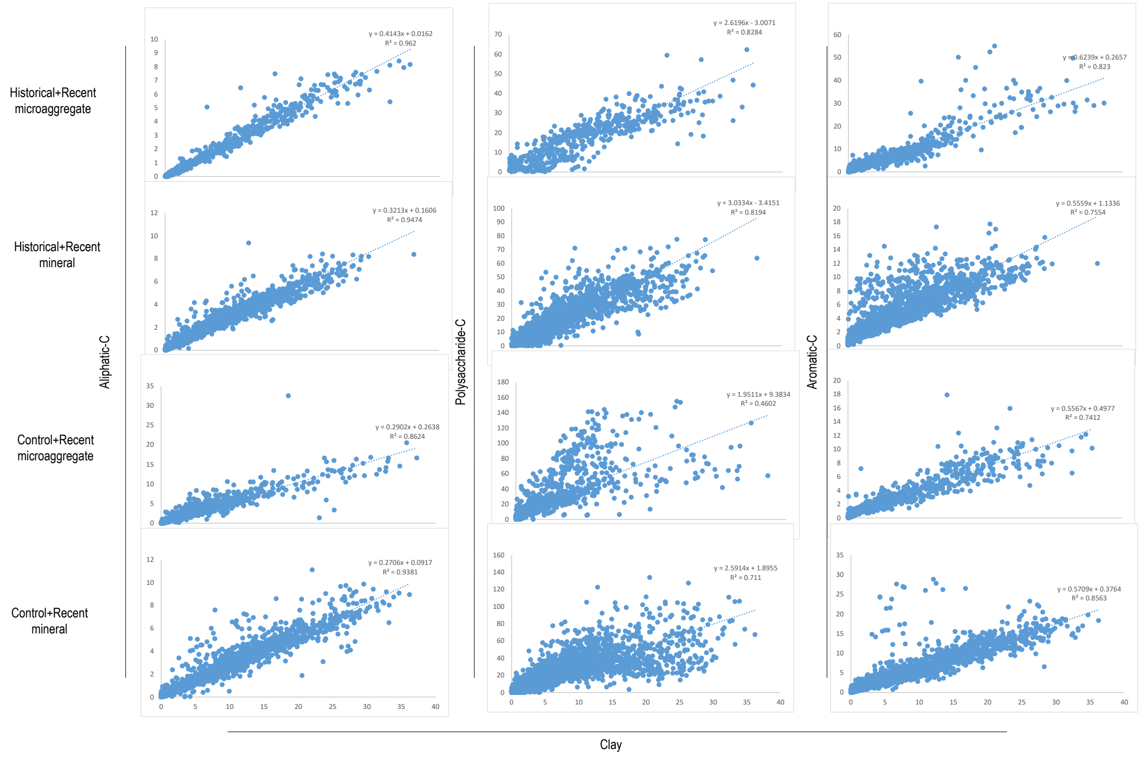

Supplementary Figure 9 | Regression analyses on a collection of spectra obtained for IRM mapping (Fig. 5b i.e., one spectrum per pixel), showing the distribution of polysaccharide-C (1035 cm^{-1}), aromatic-C (1600 cm^{-1}), aliphatic-C (2920 cm^{-1}), and mineral-OH (3650 cm^{-1}) obtained from 64 coadded scans. Only spectra where the selected absorption peak could be detected above baseline were included.

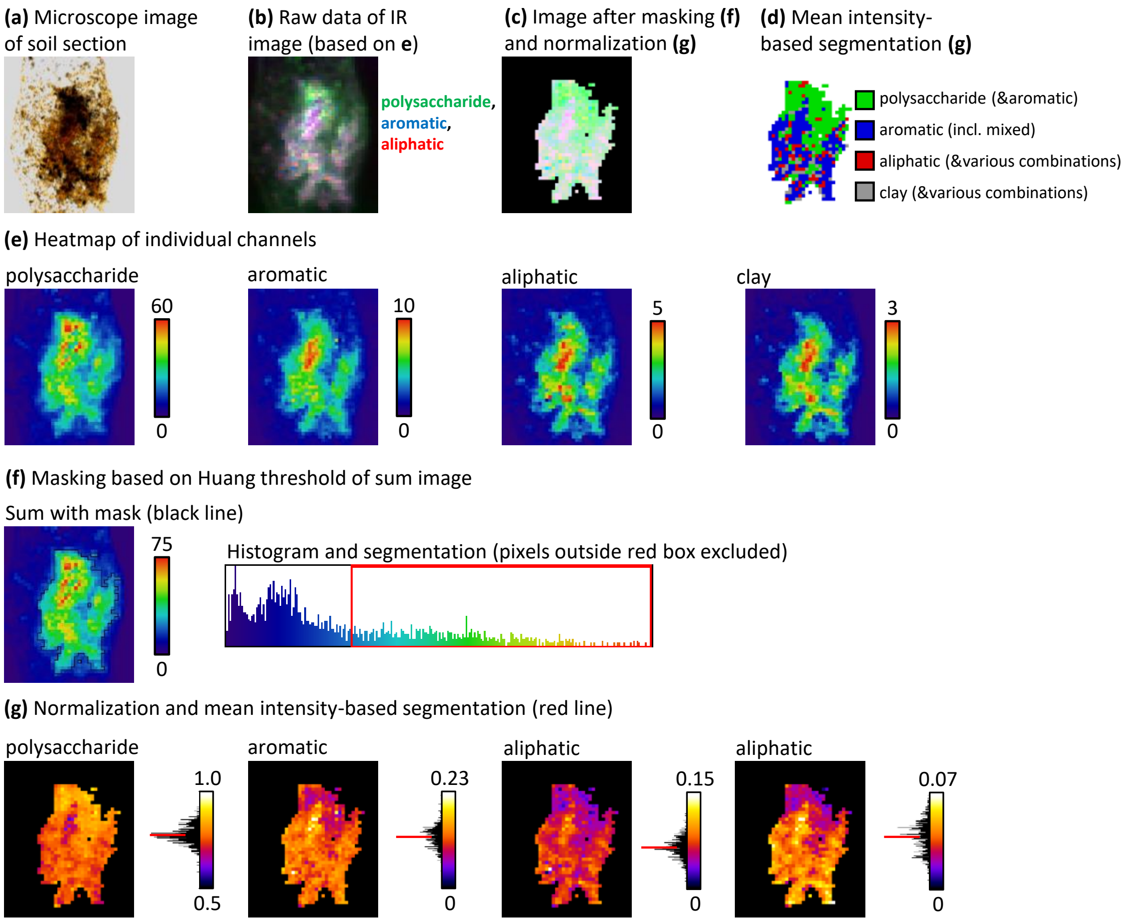

Supplementary Figure 10 | Image processing pipeline for IRM maps. a,**b**,**c,** In a given soil image (**a**), the scales of optical intensities for different organo-mineral compounds (due heterogeneous distribution and potentially varying slice thickness) were normalized across the four channels (**b**; polysaccharide-C, aromatic-C, aliphatic-C, and clay-OH) to generate a raw image (**c**). **d**, To exclude the influence of background pixels not part of the scanned soil aggregates, the histogram-based Huang thresholding algorithm (Huang and Wang 1995) was implemented in FIJI (Schindelin et al., 2015). **e**, All pixels that were part of the sample were then divided by the sum image to compute the different local proportions of the channels. Based on the individual histogram of each IR measurement, the mean value of the normalized proportion of each channel was derived and all pixels higher than that mean value were segmented as high intensity regions in a given channel. **f**,**g**, The masked, segmented images were combined to derive information about individual channels and different combinations of channels (see Supplementary Table 12, n=3).
