# Supplementary material for: Microspectroscopic visualization of how biochar lifts the soil organic carbon ceiling
Source: Nat Commun. 2022 Sep 2;13:5177. doi: 10.1038/s41467-022-32819-7 (PMC9440262; doi:10.1038/s41467-022-32819-7)
Supplement: Supplementary file 3 — Supplementary Movie1 [file 41467_2022_32819_MOESM3_ESM.pdf]

File name: Supplementary Movie 1

Description: Supplementary Movie 1: three-dimensional visualisation of rhizodeposits retention in aggregate
